# The Relationship between QT Interval Dispersion and End-Stage Liver Disease Score in the Patients Undergoing Orthotopic Liver Transplantation

**Published:** 2013-12-01

**Authors:** Moghgan Zahmatkeshan, Ebrahim Fallahzadeh, Seyedeh Sadat Najib, Hamid Amoozgar, Seyed Ali Malekhosseini, Saman Nikeghbalian

**Affiliations:** 1 Transplant Research Center, Shiraz, IR Iran; 2 Department of Internal medicine, Shiraz University of Medical Sciences, Shiraz, IR Iran; 3 Department of Pediatrics, Shiraz University of Medical Sciences, Shiraz, IR Iran; 4 Cardiovascular Research Center, Shiraz University of Medical Sciences, Shiraz, IR Iran

**Keywords:** Liver Transplantation, Electrocardiography, QT, Dispersion

## Abstract

**Background:**

This study was performed to determine the changes in corrected QT (QTc) and QT dispersion and their relationship with end-stage liver disease score in the children undergoing orthotopic liver transplantation.

**Methods:**

This case-control study was performed in a 2–year period from February 2009 to March 2011 in Department of Organ Transplantation of Nemazee Hospital. We consecutively included all the 22 pediatric patients undergoing orthotopic liver transplantation and 22 healthy age- and sex-matched controls. Electrocardiogram (ECG) was performed for all the patients and controls before and 6 months after the transplantation and the QTc was calculated according to Bazett’s formula in lead I, aVF, andV1. Besides, QT dispersion was calculated by the difference between maximum and minimum QTc in the three leads. The data were statistically analyzed using independent sample t-test, chi-square test, paired t-test, and Pearson correlation analysis. In addition, P value < 0.05 was considered as statistically significant.

**Results:**

The patients with end-stage liver disease had significantly longer QTc dispersion (P = 0.002) compared to the controls. The post-transplantation QTc dispersion (P = 0.003) was also significantly longer compared to the healthy controls. Moreover, pretransplant QTc dispersion was negatively correlated with weight (r = ‒0.589, P = 0.004) and Child-Pugh score (r = ‒0.549, P = 0.008).

**Conclusions:**

The patients with ESLD awaiting liver transplantation suffer from prolonged QTc interval predisposing them to ventricular tachycardia. The QTc prolongation in these patients does not response to liver transplantation. This study revealed a fine negative correlation between the Child- Pugh score and QTc.

## 1. Background

Liver cirrhosis and End-Stage Liver Disease (ESLD) are associated with several cardiovascular complications, including portal hypertension, central, splanchnic, and peripheral circulation abnormalities, and cardiac dysfunction and failure (1, 2) usually referred to cirrhotic cardiomyopathy (3, 4). These complications result from decreased systemic vascular resistance and ventricular contractibility leading to increased cardiac output and left ventricular ejection fraction (1). Several electrophysiological abnormalities are observed in the patients with cirrhotic cardiomyopathy the most important of which being QT-interval prolongation, chronotropic incompetence, and electromechanical uncoupling (5).

QT interval is representative of the ventricular electrical repolarization and should be corrected for the heart rate (QTc). Prolongation of the QTc (> 440 ms) is equal to prolonged repolarization period of the ventricles which in fact will result in fatal arrhythmias, including torsade de pointes associated with high mortality rate (6-8). Antiarrhythmics, certain antibiotics, ischemic heart disease, electrolyte imbalances, ion-channel disorders, and cirrhotic cardiomyopathy are known causes of acquired QTc prolongation. The prevalence of prolonged QTc in the patients with liver cirrhosis has been reported to be 56%. Although some studies have determined several independent predictors for prolonged QTc in cirrhotic patients including age, alcoholic etiology of cirrhosis, and Child-Pugh scores (9), some others have not been able to find any associations between these factors and clinical factors, such as Child-Pugh score, Model for End-Stage Liver Disease (MELD) score, Pediatric End-Stage Liver Disease (PELD) score, diabetes mellitus, age, and etiology of liver disease (10). In addition, several studies have revealed that the prevalence of prolonged QTc as well as the mean QTc interval decreased significantly after orthotopic liver transplantation (9, 10).

Currently, clinical determinates of chronic liver cirrhosis (Child-Pugh score and / or MELD / PELD scores) are used to determine the severity of the disease, predict the outcomes, assess the prognosis, and prioritize the reception of a liver transplant (11).

Although these models (MELD / PELD) are accurate in evaluating the pretransplant patients, they are not precise in determining the post-transplantation prognosis and outcome (mortality rate). Variability of the measurement of laboratory tests, including International Normalized Ratio (INR), concomitant anticoagulation usage, and coexistence of renal failure cause the MELD / PELD scores to increase falsely and, as a result, the patients with milder liver failure would be allocated to liver transplantation, while others with more severe disease will remain in the waiting list (12). Thus, adding another predictive factor, such as QTc, to the liver transplantation allocation system would increase the accuracy of clinical scoring. Hence, the present study aims to determine the relationships among MELD / PELD score, QTc, and QT dispersion in the patients undergoing orthotopic liver transplantation.

## 2. Patients and Methods

### 2.1. Study Population

This case-control study was conducted in a 2–year period from February 2009 to March 2011 in the Department of Organ Transplantation of Nemazee Hospital affiliated to Shiraz University of Medical Sciences. We consecutively included all the patients who underwent orthotopic liver transplantation in the study period. However, the patients with preexisting cardiorespiratory diseases, clinically significant valvular heart disease, septal defects, arrhythmia, interventricular conduction delay, bundle branch blocks, evidence of pre- excitation, simultaneous multiorgan transplantation, and multiple liver transplants were excluded from the study. We also included 22 healthy sex- and age-matched patients who referred to cardiology departments of Motahari Clinic of Shiraz University of Medical Sciences as the control group. None of the controls had cardiopulmonary disorders, liver failure, kidney disorders, immunologic disease, and malignancies. All the patients and controls had normal serum electrolytes, calcium, and magnesium at the time of cardiac evaluation and none of them had consumed the medications which are known to prolong repolarization 72 hours prior to cardiac evaluation. The study protocol was approved by the Institutional Review Board (IRB) and Ethics Committee of Shiraz University of Medical Sciences and all the patients and controls and their parents gave their written informed consents.

### 2.2. Study Protocol

All the patients and controls underwent physical examination and were evaluated regarding the history. Demographic information as well as the clinical findings was recorded in a data gathering form. In addition, the severity of the disease was assessed using the PELD / MELD scores (12, 13) as well as Child-Pugh classification (14). Patients were allocated to the liver transplantation according to the PELD / MELD scores. The patients’ serum levels of albumin, bilirubin, and INR were determined and the presence of ascites and hepatic encephalopathy was investigated by ultrasonography and clinical evaluation, respectively in order to determine the severity of the disease according to the clinical disseminators of liver disease (12-14).

A 12-lead electrocardiogram (ECG) was performed for all the patients and controls before the transplantation (pre-transplant ECG). All the recordings were made at a paper speed of 25 mm / s with a digital electrocardiogram machine (Alicia Diagnostics, Sanford, FL, USA). The digitally recorded electrocardiogram tracings were evaluated by a digital clipper in Corel Photo Paint v.13 software (Ottawa, Canada). Furthermore, magnification of the electrocardiogram made a fine determination of the measurement points.

The QT interval was measured from the beginning of the QRS complex to the termination of the T wave (defined as the return to the isoelectric line) in the lead I, AVF, and V1. Bazett’s formula was used to calculate the QTc: QTc = QT interval (sec) / (√R-R interval) (sec). It has been shown that QTc calculated by Bazett’s formula is an independent predictor of cardiovascular mortality (15). QTc was considered prolonged if it was > 440 ms for males and > 460 ms for females (16). It should be noted that the cut-off points of 0.44, 0.43, and 0.45 were used for 1 - 15 year old children, adult males, and adult females, respectively in order to compensate for the gender differences of QT irrespective of the liver disease. According to Locati et al. (17), females have longer QTc values compared to males. QT dispersion defined as maximum QT interval minus minimum QT interval was also calculated for each patient as an index of the spatial dispersion of ventricular recovery times. In reality, QT dispersion is a crude and approximate measure of a general abnormality of repolarization. In this study, all the patients underwent orthotopic liver transplantation with the same protocol by the same surgical team. Follow-up ECG was performed 6 months post-transplant and was interpreted in the same way mentioned above.

### 2.3. Statistical Analysis

All the statistical analyses were performed using the Statistical Package for Social Sciences version 16.0 (SPSS Inc., Chicago, Ill., USA). The results are expressed as mean ± Standard Deviation (SD) and proportions as appropriate. The quantitative data were compared between the groups using independent t-test, while chi-square test was used for comparing the proportions. Besides, paired t-test was used to determine the differences between the groups regarding QTc and QTc dispersion. Pearson correlation analysis was performed between clinical determinates of severity liver disease (MELD / PELD and Child Pugh scores) and QT. Moreover, the Kolmogorov–Smirnov test was used for evaluation of the normal distribution of the parameters. It should be mentioned that the cases and controls were matched for age ± 1 year. In addition, the power of test for measurement of QTc dispersion was 85%. A 2-sided P value of less than 0.05 was considered as statistically significant.

## 3. Results

This study was conducted on 22 patients with ESLD and 22 healthy individuals as the control group. The mean age of the patients and controls was 21.5 ± 14.4 (range 1.5 – 57) and 23.2 ± 13.5 years (range 3.2 – 41), respectively (P = 0.536). Besides, there were 16 males (72.2%) and 6 (27.3%) females among the patients and controls. Table 1 summarizes the baseline characteristics of the patients and controls. Most of the patients had high MELD / PELD scores (59.1%) and Child-Pugh class C (50.0%). Moreover, the prevalence of prolonged QTc was found to be 5 (22.7%) in the patients with ESLD. These patients had significantly longer QTc in lead I (P < 0.0001), AVF (P < 0.0001), and V1 (P < 0.0001). The QTc dispersion was also significantly longer in the patients scheduled for orthotopic liver transplantation (P = 0.002). Similarly, QTc was significantly longer 6 months after the liver transplantation in lead I (P < 0.001), AVF (P < 0.001), and V1 (P < 0.001) compared to the healthy controls. In the same way, the QTc dispersion was significantly longer even 6 months after the liver transplantation (P = 0.003) (Table 2). No significant decrease was observed in QTc in lead I (P = 0.372) and AVF (P = 0.090) and QTc dispersion (P = 0.776) 6 months after the transplantation. However, the QTc in lead V1 was significantly decreased after the transplantation (0.42 ± 0.35 vs. 0.39 ± 0.03; P = 0.004).

**Table 1.** Baseline Characteristics of 22 Patients Undergoing Liver Transplantation and 22 Healthy Age- and Sex-Matched Individuals

	Case (n = 22)	Control (n = 22)	*P* value
**Age (years)**	21.5 ± 14.4	23.2 ± 13.5	0.536
**Sex**			
**Male (%)**	16 (72%)	16 (72%)	
**Weight (kg)**	48.7 ± 24.1	47.6 ± 19.5	0.682
**Calcium**	8.3 ± 0.5	8.7 ± 0.6	0.752
**Phosphorus**	4.1 ± 1.3	4.3 ± 0.9	0.621
**Magnesium**	2.2 ± 0.4	2.8 ± 0.5	0.273
**MELD / PELD score**	22.3 ± 8.3	‒	‒
**Score ≤9**	2 (9.1%)	‒	‒
**Score 10 - 19**	7 (31.8%)	‒	‒
**Score ≥ 20**	13 (59.1%)	‒	‒
**Child-Pugh Class**	9.4 ± 2.4	‒	
**A**	2 (9.1%)	‒	‒
**B**	9 (40.9%)	‒	‒
**C**	11 (50.0%)	‒	‒
**QTc in Lead I (mm)**	0.41 ± 0.04	0.32 ± 0.03	< 0.001
**QTc in Lead AVF (mm)**	0.41 ± 0.04	0.31 ± 0.03	< 0.001
**QTc in Lead V1 (mm)**	0.42 ± 0.35	0.32 ± 0.03	< 0.001
**QTc dispersion**	0.05 ± 0.04	0.22 ± 0.02	0.002
**QT in Lead I (mm)**	0.31 ± 0.06	0.33 ± 0.02	0.167
**QT in Lead AVF (mm)**	0.32 ± 0.06	0.33 ± 0.03	0.731
**QT in Lead V1 (mm)**	0.33 ± 0.06	0.34 ± 0.03	0.594
**QT dispersion**	0.05 ± 0.03	0.02 ± 0.02	0.001

**Table 2.** QT-Segment Characteristics in 22 Patients 6 Months after Orthotopic Liver Transplantation Compared to 22 Healthy Age- and Sex-Matched Individuals

	Case (n = 22)	Control (n = 22)	*P* value
**QTc in Lead I (mm)**	0.39 ± 0.03	0.32 ± 0.03	< 0.001
**QTc in Lead AVF (mm)**	0.39 ± 0.03	0.31 ± 0.03	< 0.001
**QTc in Lead V1 (mm)**	0.39 ± 0.03	0.32 ± 0.03	< 0.001
**QTc dispersion (mm)**	0.05 ± 0.04	0.22 ± 0.02	0.003
**QT in Lead I (mm)**	0.31 ± 0.04	0.33 ± 0.03	0.176
**QT in Lead AVF (mm)**	0.31 ± 0.04	0.33 ± 0.03	0.309
**QT in Lead V1 (mm)**	0.31 ± 0.03	0.34 ± 0.03	0.005
**QT dispersion (mm)**	0.03 ± 0.01	0.02 ± 0.02	0.079

The correlation analysis between the clinical determinants of liver disease (MELD / PELD and Child-Pugh scores) and QTc dispersion revealed that pretransplant QTc dispersion was negatively correlated with weight (r = -0.589, P = 0.004) and Child-Pugh score (r = -0.549, P = 0.008). However, no linear correlation was observed between the post-transplant QTc discrepancy and MELD / PELD (r = 0.007, P = 0.974) and Child-Pugh scores (r = 0.337, P = 0.125) (Table 3). 

**Table 3.** Correlation Analysis between QTc and Clinical Determinates of Liver Disease (MELD / PELD and Child-Pugh Scores) in 22 Patients Undergoingorthotopic Liver Transplantation

	r-value	*P* value
**QTc dispersion (pretransplant)**		
**Age**	‒0.133	0.406
**Weight**	‒0.589	0.004
**Calcium**	‒0.034	0.880
**Phosphorus**	‒0.395	0.069
**Magnesium**	‒0.054	0.810
**Potassium**	‒0.128	0.571
**Sodium**	0.138	0.540
**Meld / PELD score**	‒0.332	0.131
**Child-Pugh class**	‒0.549	0.008
**QTc dispersion (post-transplant)**	‒0.108	0.632
**QTc dispersion (post-transplant)**		
**Age**	‒0.300	0.175
**Weight**	‒0.127	0.572
**Calcium**	‒0.024	0.917
**Phosphorus**	0.225	0.314
**Magnesium**	‒0.305	0.168
**Potassium**	‒0.376	0.085
**Sodium**	0.007	0.976
**Meld / PELD score**	0.007	0.974
**Child-Pugh class**	0.337	0.125

## 4. Discussion

The cardiac electrophysiology abnormalities in cirrhotic patients and those with ESLD are well established and documented. Although the mechanism of these cardiac abnormalities is not fully understood yet, it is postulated that these alterations are because of molecular alterations of conductive system and myocardial muscles of the heart (5). Another important factor in determining the electrocardiographic abnormalities of cirrhotic patients is to eliminate the other causes of these disturbances, including ischemia, valvular heart disease, restrictive or congenital heart disease, conduction abnormalities, arterial hypertension, lung disease, using anti-arrhythmic drugs, and active consumption of alcoholic beverages. Up to now, QTc interval prolongation has been documented in the patients with chronic liver disease. In this study, we tried to determine the length of QTc and QT dispersion in the patients awaiting orthotopic liver transplantation and compare them with healthy individuals. We also investigated the variations of the QTc after orthotopic liver transplantation in order to be able to comment on the role of liver replacement therapy in treating these cardiac abnormalities.

The findings of the present study showed that QTc and QTc dispersion were prolonged in the patients with ESLD both before and after the liver transplantation compared to the healthy individuals, demonstrating the irreversible nature of the cardiac electrophysiological changes. These findings are consistent with the previous reports showing a 50% prevalence of prolonged QTc in the patients with severe liver disease (8). Most studies have found the prevalence of prolonged QTc to range from 36% to 60% in ESLD patients (9, 10, 18). However, one study conducted on 37 subjects in Spain did not have any enrollees with a QTc > 440 msec and, conversely, the prevalence of prolonged QTc (> 440 ms) was 87% in a study performed in Birmingham, England (19, 20). Zurick and colleagues (8) also showed that the QTc interval was significantly shortened post liver transplantation which is on the contrary to our results. Shin et al. (21) showed that about half of the patients awaiting liver transplantation suffered from prolonged QTc. However, they found that prolonged QTc did not return to the baseline level in the neohepatic stages even among the patients with baseline QTc < 440 msec. In addition, about half of the patients experienced a marked prolongation of QTc ≥ 500 msec in the anhepatic stage returning to baseline in the neohepatic stage.

In cirrhotic patients, the exact mechanism of QTc prolongation is yet to be identified. Up to now, several factors, including electrolyte abnormalities, myocardial ischemia, and sympathetic hyperactivity, have been identified to be associated with QTc prolongation in these patients. These factors can even be aggravated during the operation because of hypovolemia secondary to massive bleeding during the dissection and compression of the inferior vena cava in the anhepatic stage. However, it was shown by Shin et al. (21) that the QTc was remained prolonged throughout the liver transplantation procedure even in the patients with baseline QTc < 440 msec probably because of sympathetic hyperactivity and drugs affecting QTc interval during the procedure (20). It has been reported that plasma norepinephrine concentration peaks coincide with the start of the anhepatic and the neohepatic stages (22). Moreover, the drugs used as anesthetics during the operation, including isoflurane, fentanyl, and vecuronium, affect the QTc interval (23).

In this study, we tried to determine the correlation between the clinical indices of chronic liver disease (MELD / PELD and Child Pugh scores) and the QTc prolongation. However, no associations were found among these parameters, except for a negative linear correlation between pre-transplantation QTc dispersion and the Child Pugh score. Nevertheless, QT interval is recommended to be used as a complimentary method to predict the cardiovascular outcomes post transplantation.

Previous reports also have revealed no associations between the pre-transplant QTc interval and various clinical variables (10, 20). Some previous studies have indicated an association between Child-Pugh or MELD score and the QTc interval, but the majority of studies have not shown any correlation. In agreement with the previous reports, our study confirmed the lack of associations among MELD / PELD score, Child- Pugh score, and the QTc interval (8). This can perhaps be explained by the lack of any association between these various clinical variables and autonomic function. MELD was originally used to determine the priority of organ transplant recipients (24). It was later modified to estimate the severity of ESLD in pre-transplant patients; increased MELD score was closely associated with increased mortality (11). MELD / PELD is an accurate model for evaluating the pre-transplant conditions of the cirrhotic patients; however, it is not accurate in predicting the prognosis and mortality mainly because of variability of the measurement of laboratory tests, including International Normalized Ratio (INR), concomitant anticoagulation usage, and coexistence of renal failure (12). Our study showed a fine correlation between QT dispersion and Child-Pugh score.

We note some limitations in our study with the small study population being the main one. The results of this study are limited by the small number of patients. However, the follow-up period was appropriate, while other authors (8) have postulated that 1 month is insufficient to allow the QT to reach its new baseline following liver transplantation. One other important limitation is that all the pre-transplant ECGs were performed in a specific time period with the significant electrolyte alterations typically encountered in the ESLD patient population that may have resulted in bias of the confounding factors. However, we measured the serum levels of important electrolytes in order to compensate for these factors.

In conclusion, the patients with ESLD awaiting liver transplantation suffer from prolonged QTc and QT dispersion interval predisposing them to ventricular tachycardia. The QTc and QT dispersion prolongation in these patients does not response liver transplantation. Thus, the risk does not decrease even after the transplantation and may even worsen the post-transplant outcome of these patients.

### Study Limitations

Because of the small number of patients, our study should be considered as a preliminary report, and we recommend further research in this field.
